# [4-(2-Amino­ethyl)piperazin-1-ium]trichloridocopper(II) monohydrate

**DOI:** 10.1107/S1600536809046121

**Published:** 2009-11-07

**Authors:** Baccar Ikram, Zouari Fatma

**Affiliations:** a Laboratoire de Sciences de Matériaux et d’Environnement, Faculté des Sciences de SFAX, BP 802, 3018 SFAX, Tunisia

## Abstract

In the title compound, [CuCl_3_(C_6_H_16_N_3_)]·H_2_O, the copper(II) ion is five-coordinated by two N atoms from the bidentate 4-(2-amino­ethyl)piperazin-1-ium cation and three chloride ions in a distorted square-pyramidal environment. Inter­molecular N—H⋯Cl and O—H⋯Cl hydrogen bonds build up an intricate three-dimensional network.

## Related literature

For background information on polydentate ligands with nitro­gen donor atoms, see: Riggio *et al.* (2001 ▶); Xiang *et al.* (2007 ▶); Gokhale *et al.* (2001 ▶). The copper(II) ion, owing to the ’plasticity’ of the coordination sphere, forms complexes of coordination number 4–6, with a variety of irregular geometries, see: Fujisawa *et al.* (2008 ▶).
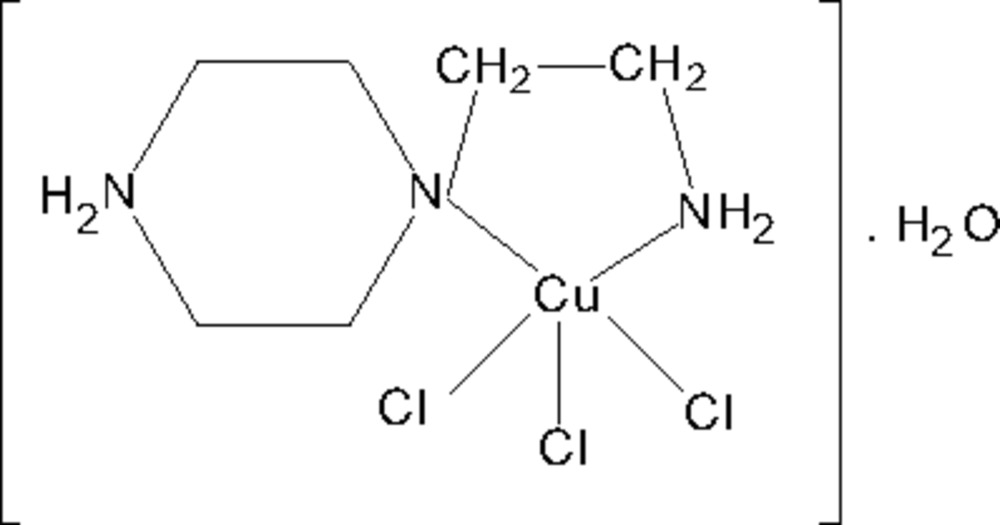


## Experimental

### 

#### Crystal data

[CuCl_3_(C_6_H_16_N_3_)]·H_2_O
*M* *_r_* = 318.13
Monoclinic, 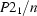

*a* = 9.0540 (6) Å
*b* = 14.8840 (13) Å
*c* = 9.1040 (2) Å
β = 94.019 (5)°
*V* = 1223.84 (14) Å^3^
*Z* = 4
Mo *K*α radiation
μ = 2.41 mm^−1^
*T* = 293 K
0.35 × 0.21 × 0.15 mm

#### Data collection

Bruker SMART CCD area-detector diffractometer
Absorption correction: multi-scan (*SADABS*; Bruker *et al.*, 1998 ▶) *T*_min_ = 0.541, *T*_max_ = 0.685
21677 measured reflections
4410 independent reflections
3444 reflections with *I* > 2σ(*I*)
*R*_int_ = 0.032

#### Refinement

*R*[*F* ^2^ > 2σ(*F* ^2^)] = 0.038
*wR*(*F* ^2^) = 0.102
*S* = 1.19
4410 reflections
127 parameters
H-atom parameters constrained
Δρ_max_ = 0.75 e Å^−3^
Δρ_min_ = −0.73 e Å^−3^

### 

Data collection: *SMART* (Bruker, 1998 ▶); cell refinement: *SAINT-Plus* (Bruker, 1998 ▶); data reduction: *SAINT-Plus*; program(s) used to solve structure: *SHELXS97* (Sheldrick, 2008 ▶); program(s) used to refine structure: *SHELXL97* (Sheldrick, 2008 ▶); molecular graphics: *ORTEPIII* (Burnett & Johnson, 1996 ▶), *ORTEP-3 for Windows* (Farrugia, 1997 ▶) and *PLATON* (Spek, 2009 ▶); software used to prepare material for publication: *WinGX* (Farrugia, 1999 ▶).

## Supplementary Material

Crystal structure: contains datablocks I, global. DOI: 10.1107/S1600536809046121/dn2503sup1.cif

Structure factors: contains datablocks I. DOI: 10.1107/S1600536809046121/dn2503Isup2.hkl

Additional supplementary materials:  crystallographic information; 3D view; checkCIF report

## Figures and Tables

**Table 1:** Hydrogen-bond geometry (Å, °)

*D*—H⋯*A*	*D*—H	H⋯*A*	*D*⋯*A*	*D*—H⋯*A*
O1*W*—H1⋯Cl2	0.97	2.21	3.154 (4)	164
O1*W*—H2⋯Cl1^i^	0.97	2.40	3.239 (4)	144
N1—H1*A*⋯Cl1^ii^	0.90	2.63	3.395 (2)	143
N1—H1*B*⋯Cl3^iii^	0.90	2.43	3.302 (2)	162
N3—H3*A*⋯Cl2^iv^	0.90	2.38	3.204 (2)	152
N3—H3*B*⋯Cl3^v^	0.90	2.24	3.131 (2)	169

Symmetry codes: (i) 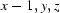; (ii) 
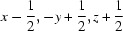
; (iii) 
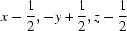
; (iv) 
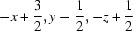
; (v) 
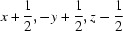
.

**Fig. 1.**
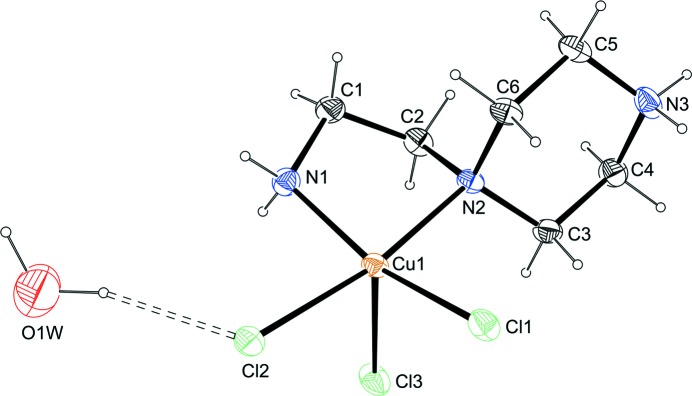
Molecular view of the title compound, showing the atom-numbering scheme. Displacement ellipsoids are drawn at 30% probabilit level. H atoms are represented as small spheres of arbitrary radii. H bond is shown as dashed line.

**Fig. 2.**
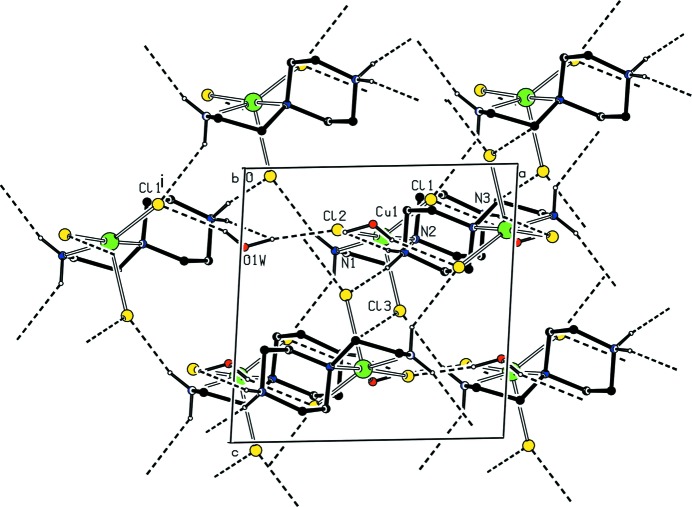
Partial packing view showing the intricated N—H···Cl and O—H···Cl network. H atoms not involved in hydrogen bondings have been omitted for clarity. H bonds are shown as dashed lines. [Symmetry code: (i) x - 1, y, z]

## supplementary crystallographic information

### Comment

Polydentate ligands with nitrogen donor atoms are largely employed in mimicking the environnement of the copper in models of biological interest, whose coordination environment is provided by nitrogen atoms of the ligand, plus one or more exogeneous ligands (Riggio *et al.*, 2001; Xiang *et al.*, 2007; Gokhale *et al.*, 2001). It is of current interest to correlate the flexibility of the model ligand and its sterical hindrance with the geometry of the complex. The copper(II) ion, owing to the well known 'plasticity' of the coordination sphere, forms complexes of co-ordination number 4–6, with a variety of irregular geometries (Fujisawa *et al.*, 2008), both the anions and the solvent playing often a vital role on the stoichiometry and stereochemistry of the complexes (Xiang *et al.*, 2007). In this paper, we report on the synthesis and the crystal structure determinationof N2 bidentate copper(II) complex 1-(2-ammoniumethyl) piperazinium trichlorocuprate(II)monohydrate (Scheme).

The structure of the title compound consists of discrete copper(II) neutral complexes with the metal atom five-coordinated to two N atoms from the bidentate 1-(2-aminoethyl) piperazine and three chlorine atoms in a square pyramidal environment (Fig. 1). The square plane is defined by two N atoms from organic cation and the more strongly bonded Cl1 and Cl2 chlorine, the apical position is occupied by the Cl3 chlorine atom. The Cu—Cl3 distance 2.6095 (8)Å is significantly longer than the normal bond length, which reflects the weak axial interactions as expected for Jahn-Teller distorted copper (II) complexes. The value of structural parameter τ is 0.17, showing a distorted square pyramidal structure, where τ is defined as τ = (α-β)/60°, where α and β are the largest angles (α> β) around a five coordinated metal center (τ is equal to 0 for an ideal square pyramidal geometry).

The intermolecular hydrogen bonds, N—H···Cl and O—H···Cl, build up an intricated three dimensionnal network (Table 1, Fig. 2).

### Experimental

To a solution of 1-(2-aminoethyl) piperazine compound (10 mmol) in chlorhydric acid (35 ml) was added a solution of CuCl_2_.2H_2_O(10 mmol) in acetone (15 ml). After a few days to 3 weeks, the product separates as crystals, which were isolated by filtration and dried in air. The new compound show satisfactory elemental analyses.

### Refinement

All H atoms attached to C atoms and N atom were fixed geometrically and treated as riding with C—H = 0.97 Å and N—H = 0.90 Å with *U*_iso_(H) = 1.2*U*_eq_(C,*N*). H atoms of water molecule were located in difference Fourier maps and included in the subsequent refinement using restraints (O—H= 0.85 (1)Å and H···H= 1.39 (2) Å) with *U*_iso_(H) = 1.5*U*_eq_(O). In the last stages of refinement, they were treated as riding on the O atom.

### Crystal data

**Table:**

[CuCl_3_(C_6_H_16_N_3_)]·H_2_O	*F*(000) = 652
*M**_r_* = 318.13	*D*_x_ = 1.727 Mg m^−^^3^
Monoclinic, *P*2_1_/*n*	Melting point: 455 K
Hall symbol: -P 2yn	Mo *K*α radiation, λ = 0.71073 Å
*a* = 9.0540 (6) Å	Cell parameters from 4604 reflections
*b* = 14.8840 (13) Å	θ = 3.1–33.0°
*c* = 9.1040 (2) Å	µ = 2.41 mm^−^^1^
β = 94.019 (5)°	*T* = 293 K
*V* = 1223.84 (14) Å^3^	Prism, colourless
*Z* = 4	0.35 × 0.21 × 0.15 mm

### Data collection

**Table:**

Bruker SMART CCD area-detector diffractometer	4410 independent reflections
Radiation source: fine-focus sealed tube	3444 reflections with *I* > 2σ(*I*)
graphite	*R*_int_ = 0.032
φ and ω scans	θ_max_ = 33.0°, θ_min_ = 3.1°
Absorption correction: multi-scan (*SADABS*; Bruker, 1998)	*h* = −13→13
*T*_min_ = 0.541, *T*_max_ = 0.685	*k* = −20→22
21677 measured reflections	*l* = −13→13

### Refinement

**Table:**

Refinement on *F*^2^	Primary atom site location: structure-invariant direct methods
Least-squares matrix: full	Secondary atom site location: difference Fourier map
*R*[*F*^2^ > 2σ(*F*^2^)] = 0.038	Hydrogen site location: inferred from neighbouring sites
*wR*(*F*^2^) = 0.102	H-atom parameters constrained
*S* = 1.19	*w* = 1/[σ^2^(*F*_o_^2^) + (0.0308*P*)^2^ + 1.5885*P*] where *P* = (*F*_o_^2^ + 2*F*_c_^2^)/3
4410 reflections	(Δ/σ)_max_ < 0.001
127 parameters	Δρ_max_ = 0.75 e Å^−^^3^
0 restraints	Δρ_min_ = −0.73 e Å^−^^3^

### Special details

**Table:**

Geometry. All e.s.d.'s (except the e.s.d. in the dihedral angle between two l.s. planes) are estimated using the full covariance matrix. The cell e.s.d.'s are taken into account individually in the estimation of e.s.d.'s in distances, angles and torsion angles; correlations between e.s.d.'s in cell parameters are only used when they are defined by crystal symmetry. An approximate (isotropic) treatment of cell e.s.d.'s is used for estimating e.s.d.'s involving l.s. planes.
Refinement. Refinement of *F*^2^ against ALL reflections. The weighted *R*-factor *wR* and goodness of fit *S* are based on *F*^2^, conventional *R*-factors *R* are based on *F*, with *F* set to zero for negative *F*^2^. The threshold expression of *F*^2^ > σ(*F*^2^) is used only for calculating *R*-factors(gt) *etc*. and is not relevant to the choice of reflections for refinement. *R*-factors based on *F*^2^ are statistically about twice as large as those based on *F*, and *R*- factors based on ALL data will be even larger.

### Fractional atomic coordinates and isotropic or equivalent isotropic displacement parameters (Å^2^)

**Table:**

	*x*	*y*	*z*	*U*_iso_*/*U*_eq_	
Cu1	0.52502 (3)	0.275723 (19)	0.26008 (3)	0.02535 (8)	
Cl1	0.69453 (8)	0.34628 (4)	0.12625 (7)	0.03484 (14)	
Cl2	0.35774 (8)	0.39523 (4)	0.23087 (8)	0.03722 (15)	
Cl3	0.60236 (8)	0.32232 (5)	0.53095 (8)	0.04205 (17)	
N1	0.3558 (2)	0.19697 (14)	0.3082 (2)	0.0301 (4)	
H1A	0.3237	0.2139	0.3954	0.036*	
H1B	0.2807	0.2044	0.2391	0.036*	
N2	0.6475 (2)	0.15459 (12)	0.2700 (2)	0.0238 (4)	
N3	0.8947 (3)	0.04353 (15)	0.1831 (3)	0.0373 (5)	
H3A	0.9412	−0.0099	0.1866	0.045*	
H3B	0.9465	0.0810	0.1285	0.045*	
C1	0.3973 (3)	0.10108 (17)	0.3155 (3)	0.0339 (5)	
H11	0.3787	0.0734	0.2196	0.041*	
H12	0.3389	0.0699	0.3850	0.041*	
C2	0.5603 (3)	0.09485 (16)	0.3645 (3)	0.0305 (5)	
H21	0.5758	0.1131	0.4667	0.037*	
H22	0.5936	0.0332	0.3565	0.037*	
C3	0.8026 (3)	0.16627 (16)	0.3349 (3)	0.0308 (5)	
H31	0.7998	0.1878	0.4352	0.037*	
H32	0.8515	0.2117	0.2792	0.037*	
C4	0.8927 (3)	0.08025 (18)	0.3355 (3)	0.0353 (6)	
H41	0.9932	0.0923	0.3744	0.042*	
H42	0.8500	0.0362	0.3987	0.042*	
C5	0.7427 (3)	0.03183 (17)	0.1101 (3)	0.0344 (6)	
H51	0.6915	−0.0157	0.1589	0.041*	
H52	0.7497	0.0147	0.0081	0.041*	
C6	0.6557 (3)	0.11889 (17)	0.1175 (3)	0.0297 (5)	
H61	0.7012	0.1639	0.0583	0.036*	
H62	0.5559	0.1089	0.0748	0.036*	
O1W	0.0185 (4)	0.3850 (3)	0.2878 (5)	0.0994 (12)	
H1	0.1193	0.3767	0.2606	0.149*	
H2	−0.0540	0.3497	0.2295	0.149*	

### Atomic displacement parameters (Å^2^)

**Table:**

	*U*^11^	*U*^22^	*U*^33^	*U*^12^	*U*^13^	*U*^23^
Cu1	0.02915 (15)	0.02000 (13)	0.02711 (15)	0.00087 (10)	0.00348 (10)	0.00021 (10)
Cl1	0.0433 (4)	0.0240 (3)	0.0390 (3)	0.0025 (2)	0.0153 (3)	0.0055 (2)
Cl2	0.0342 (3)	0.0270 (3)	0.0499 (4)	0.0043 (2)	−0.0007 (3)	−0.0008 (3)
Cl3	0.0426 (4)	0.0499 (4)	0.0325 (3)	0.0124 (3)	−0.0058 (3)	−0.0154 (3)
N1	0.0291 (10)	0.0298 (10)	0.0315 (10)	−0.0012 (8)	0.0021 (8)	0.0019 (8)
N2	0.0308 (10)	0.0188 (8)	0.0220 (8)	0.0011 (7)	0.0029 (7)	0.0017 (6)
N3	0.0422 (13)	0.0230 (9)	0.0489 (14)	0.0071 (9)	0.0190 (11)	0.0034 (9)
C1	0.0362 (13)	0.0263 (11)	0.0402 (14)	−0.0064 (10)	0.0097 (11)	0.0001 (10)
C2	0.0397 (14)	0.0253 (10)	0.0271 (11)	0.0018 (9)	0.0071 (10)	0.0056 (9)
C3	0.0340 (13)	0.0228 (10)	0.0352 (13)	0.0000 (9)	−0.0007 (10)	−0.0031 (9)
C4	0.0335 (13)	0.0278 (11)	0.0440 (15)	0.0056 (10)	−0.0023 (11)	0.0010 (10)
C5	0.0496 (16)	0.0239 (11)	0.0311 (12)	−0.0004 (10)	0.0128 (11)	−0.0036 (9)
C6	0.0418 (14)	0.0271 (11)	0.0204 (10)	0.0006 (10)	0.0035 (9)	−0.0004 (8)
O1W	0.072 (2)	0.120 (3)	0.105 (3)	−0.002 (2)	0.002 (2)	−0.018 (2)

### Geometric parameters (Å, °)

**Table:**

Cu1—N1	2.002 (2)	C1—H11	0.9700
Cu1—N2	2.1153 (19)	C1—H12	0.9700
Cu1—Cl1	2.2814 (7)	C2—H21	0.9700
Cu1—Cl2	2.3392 (7)	C2—H22	0.9700
Cu1—Cl3	2.6097 (7)	C3—C4	1.518 (4)
N1—C1	1.476 (3)	C3—H31	0.9700
N1—H1A	0.9000	C3—H32	0.9700
N1—H1B	0.9000	C4—H41	0.9700
N2—C6	1.493 (3)	C4—H42	0.9700
N2—C3	1.495 (3)	C5—C6	1.520 (4)
N2—C2	1.500 (3)	C5—H51	0.9700
N3—C4	1.493 (4)	C5—H52	0.9700
N3—C5	1.496 (4)	C6—H61	0.9700
N3—H3A	0.9000	C6—H62	0.9700
N3—H3B	0.9000	O1W—H1	0.9701
C1—C2	1.514 (4)	O1W—H2	0.9694
			
N1—Cu1—N2	84.19 (8)	H11—C1—H12	108.4
N1—Cu1—Cl1	160.09 (7)	N2—C2—C1	109.6 (2)
N2—Cu1—Cl1	92.56 (6)	N2—C2—H21	109.7
N1—Cu1—Cl2	88.32 (7)	C1—C2—H21	109.7
N2—Cu1—Cl2	170.50 (6)	N2—C2—H22	109.7
Cl1—Cu1—Cl2	92.50 (3)	C1—C2—H22	109.7
N1—Cu1—Cl3	96.18 (7)	H21—C2—H22	108.2
N2—Cu1—Cl3	94.65 (6)	N2—C3—C4	113.1 (2)
Cl1—Cu1—Cl3	103.67 (3)	N2—C3—H31	109.0
Cl2—Cu1—Cl3	91.95 (3)	C4—C3—H31	109.0
C1—N1—Cu1	112.31 (16)	N2—C3—H32	109.0
C1—N1—H1A	109.1	C4—C3—H32	109.0
Cu1—N1—H1A	109.1	H31—C3—H32	107.8
C1—N1—H1B	109.1	N3—C4—C3	110.3 (2)
Cu1—N1—H1B	109.1	N3—C4—H41	109.6
H1A—N1—H1B	107.9	C3—C4—H41	109.6
C6—N2—C3	107.62 (19)	N3—C4—H42	109.6
C6—N2—C2	112.64 (19)	C3—C4—H42	109.6
C3—N2—C2	111.08 (19)	H41—C4—H42	108.1
C6—N2—Cu1	108.90 (14)	N3—C5—C6	110.1 (2)
C3—N2—Cu1	113.13 (14)	N3—C5—H51	109.6
C2—N2—Cu1	103.53 (14)	C6—C5—H51	109.6
C4—N3—C5	112.6 (2)	N3—C5—H52	109.6
C4—N3—H3A	109.1	C6—C5—H52	109.6
C5—N3—H3A	109.1	H51—C5—H52	108.1
C4—N3—H3B	109.1	N2—C6—C5	113.8 (2)
C5—N3—H3B	109.1	N2—C6—H61	108.8
H3A—N3—H3B	107.8	C5—C6—H61	108.8
N1—C1—C2	108.2 (2)	N2—C6—H62	108.8
N1—C1—H11	110.1	C5—C6—H62	108.8
C2—C1—H11	110.1	H61—C6—H62	107.7
N1—C1—H12	110.1	H1—O1W—H2	113.8
C2—C1—H12	110.1		

### Hydrogen-bond geometry (Å, °)

**Table:**

*D*—H···*A*	*D*—H	H···*A*	*D*···*A*	*D*—H···*A*
O1W—H1···Cl2	0.97	2.21	3.154 (4)	164
O1W—H2···Cl1^i^	0.97	2.40	3.239 (4)	144
N1—H1A···Cl1^ii^	0.90	2.63	3.395 (2)	143
N1—H1B···Cl3^iii^	0.90	2.43	3.302 (2)	162
N3—H3A···Cl2^iv^	0.90	2.38	3.204 (2)	152
N3—H3B···Cl3^v^	0.90	2.24	3.131 (2)	169

Symmetry codes: (i) *x*−1, *y*, *z*; (ii) *x*−1/2, −*y*+1/2, *z*+1/2; (iii) *x*−1/2, −*y*+1/2, *z*−1/2; (iv) −*x*+3/2, *y*−1/2, −*z*+1/2; (v) *x*+1/2, −*y*+1/2, *z*−1/2.
